# MR imaging features to predict the type of bone metastasis in prostate cancer

**DOI:** 10.1038/s41598-023-38878-0

**Published:** 2023-07-18

**Authors:** Hiroaki Koyama, Ryo Kurokawa, Shimpei Kato, Masanori Ishida, Ryohei Kuroda, Tetsuo Ushiku, Haruki Kume, Osamu Abe

**Affiliations:** 1 Department of Radiology, Graduate School of Medicine, The University of Tokyo, 7-3-1 Hongo, Bunkyo-ku, Tokyo 113-8655 Japan; 2 Department of Pathology, Graduate School of Medicine, The University of Tokyo, 7-3-1 Hongo, Bunkyo-ku, Tokyo 113-8655 Japan; 3 Department of Urology, Graduate School of Medicine, The University of Tokyo, 7-3-1 Hongo, Bunkyo-ku, Tokyo 113-8655 Japan

**Keywords:** Cancer imaging, Bone metastases, Prostate cancer

## Abstract

Bone metastases (BMs) of prostate cancer (PCa) have been considered predominantly osteoblastic, but non-osteoblastic (osteolytic or mixed osteoblastic and osteolytic) BMs can occur. We investigated the differences in prostate MRI and clinical findings between patients with osteoblastic and non-osteoblastic BMs. Between 2014 and 2021, patients with pathologically proven PCa without a history of other malignancies were included in this study. Age, Gleason score, prostate-specific antigen (PSA) density, normalized mean apparent diffusion coefficient and normalized T2 signal intensity (nT2SI) of PCa, and Prostate Imaging Reporting and Data System category on MRI were compared between groups. A multivariate logistic regression analysis using factors with *P*-values < 0.2 was performed to detect the independent parameters for predicting non-osteoblastic BM group. Twenty-five (mean 73 ± 6.6 years) and seven (69 ± 13.1 years) patients were classified into the osteoblastic and non-osteoblastic groups, respectively. PSA density and nT2SI were significantly higher in the non-osteoblastic group than in the osteoblastic group. nT2SI was an independent predictive factor for non-osteoblastic BMs in the multivariate logistic regression analysis. These results indicated that PCa patients with high nT2SI and PSA density should be examined for osteolytic BMs.

## Introduction

Prostate cancer (PCa) is the most frequently diagnosed cancer in men in 112 of 185 countries, with almost 1.4 million new patients diagnosed and 375,000 deaths worldwide in 2020^1^. Bone metastases (BMs) are present in approximately 3% of patients at the time of diagnosis, and 11.5% of patients are diagnosed with BMs during follow-up^2^. BMs of PCa can cause skeletal complications, such as pain, pathologic fracture, and spinal cord compression, which adversely affect quality of life^3^. The BMs of PCa have been considered predominantly osteoblastic, and therefore, osteolytic lesions could be attributed to other causes, such as multiple myeloma and metastases from other malignancies (e.g., hepatocellular carcinoma, renal cell carcinoma, and thyroid cancer)^4^. However, some PCa develop osteolytic or mixed (both osteoblastic and osteolytic) BMs, with 72.9% reported as osteoblastic, 9.5% as osteolytic, and 17.6% as mixed^5^.

Prostate MRI has been useful for detecting clinically significant PCa, and the Prostate Imaging Reporting and Data System (PI-RADS)^6^ has been widely used in clinical practice as a tool for risk stratification and promoting standardized reporting^7–9^. The sensitivity and specificity of PI-RADS v2.1 for clinically significant PCa are reported to be 97% (95% confidence intervals, 82–91%) and 74% (63–82%), respectively^8^. Prostate MRI is also useful for assessing local progression, which influences treatment strategies^10,11^. Since it has been reported that osteolytic BMs are not rare in high-risk PCa^12^ and that high-risk PCa tends to show a low apparent diffusion coefficient (ADC) and low T2 signal intensity^13–18^, we hypothesized that the MR signals of PCa would also differ depending on the type of BMs. However, the relationship between prostate MRI features and BM type has not been elucidated, and this knowledge would be helpful to infer the BM type from widely used prostate MRI findings.

The purpose of this study was to investigate the differences in pretreatment MRI and clinical findings between patients with osteoblastic and non-osteoblastic (osteolytic or mixed) BMs.

## Results

**Figure 1:**
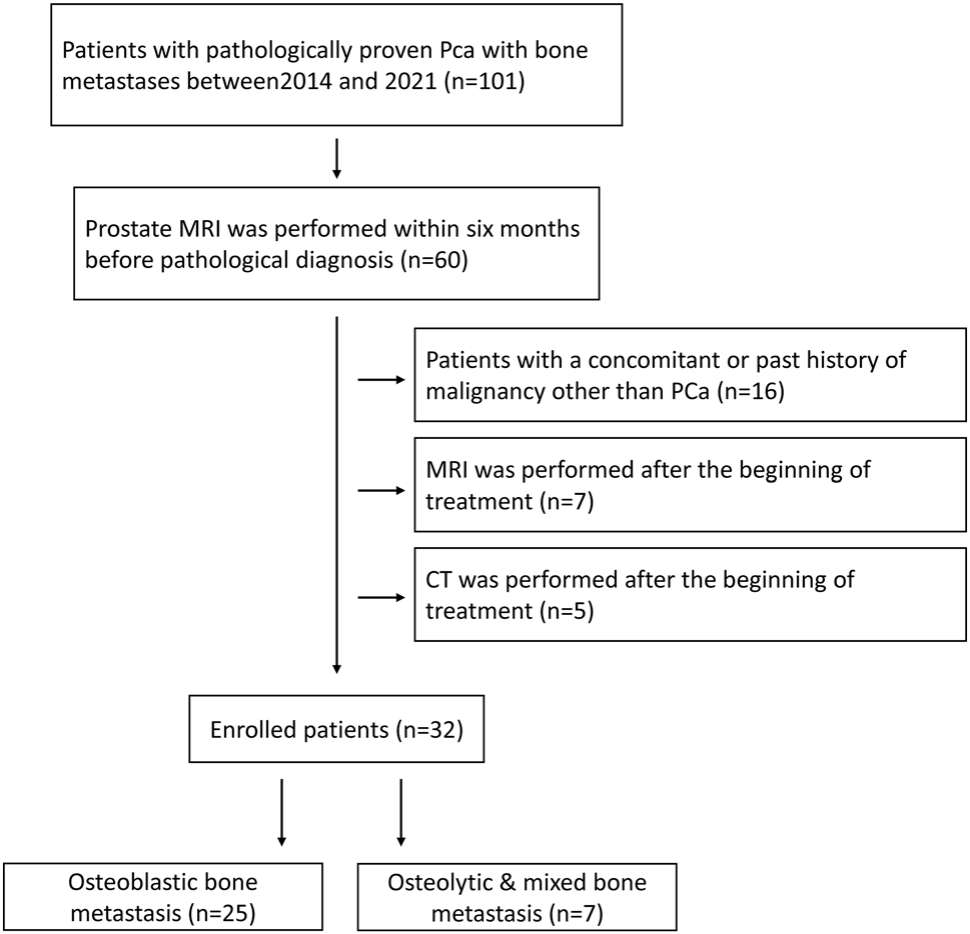
Study flow chart.

Of the 2314 patients diagnosed with PCa between 2014 and 2021, 101 had BMs, and 60 patients received prostate MRI within six months before pathological diagnosis. After excluding patients with a history of malignancy other than PCa (n = 16) and those for whom treatment had already started at the time of MRI (n = 7) and CT (n = 5), 32 patients were included in the present study (Fig. 1). Twenty-five (mean age, 73 ± 6.6 years) and seven (mean age, 69 ± 13.1 years) patients were classified into the osteoblastic and non-osteoblastic groups, respectively. Two and five patients in the non-osteoblastic group had osteolytic and mixed BMs, respectively.

### Clinical and radiological analyses

**Figure 2:**
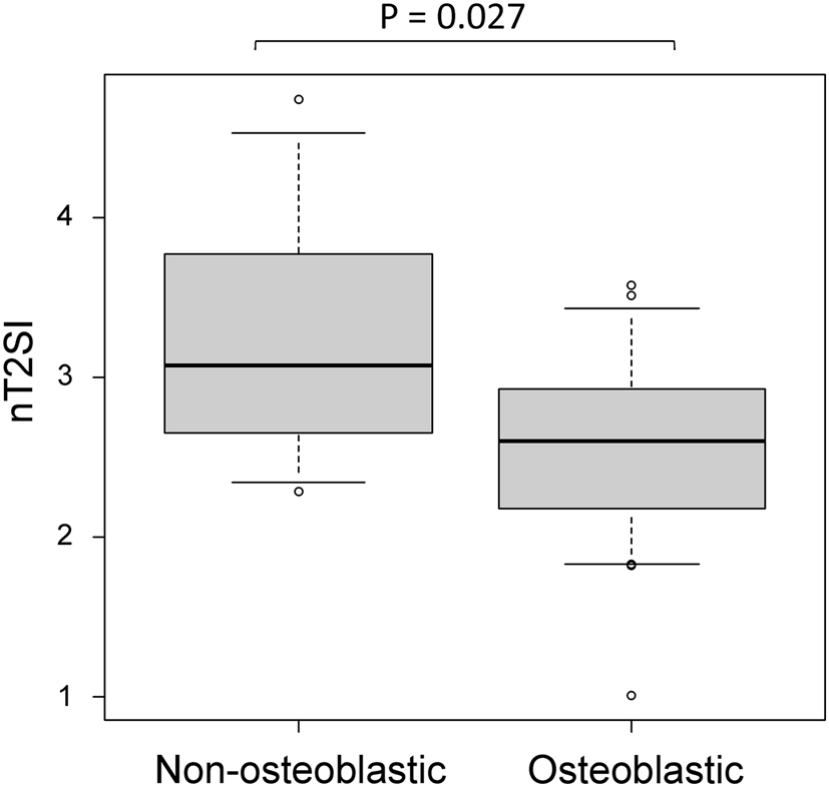
Comparison of nT2SI between the osteoblastic and non-osteoblastic groups. The median and 10th and 90th percentiles are marked with boxplots.

**Figure 3:**
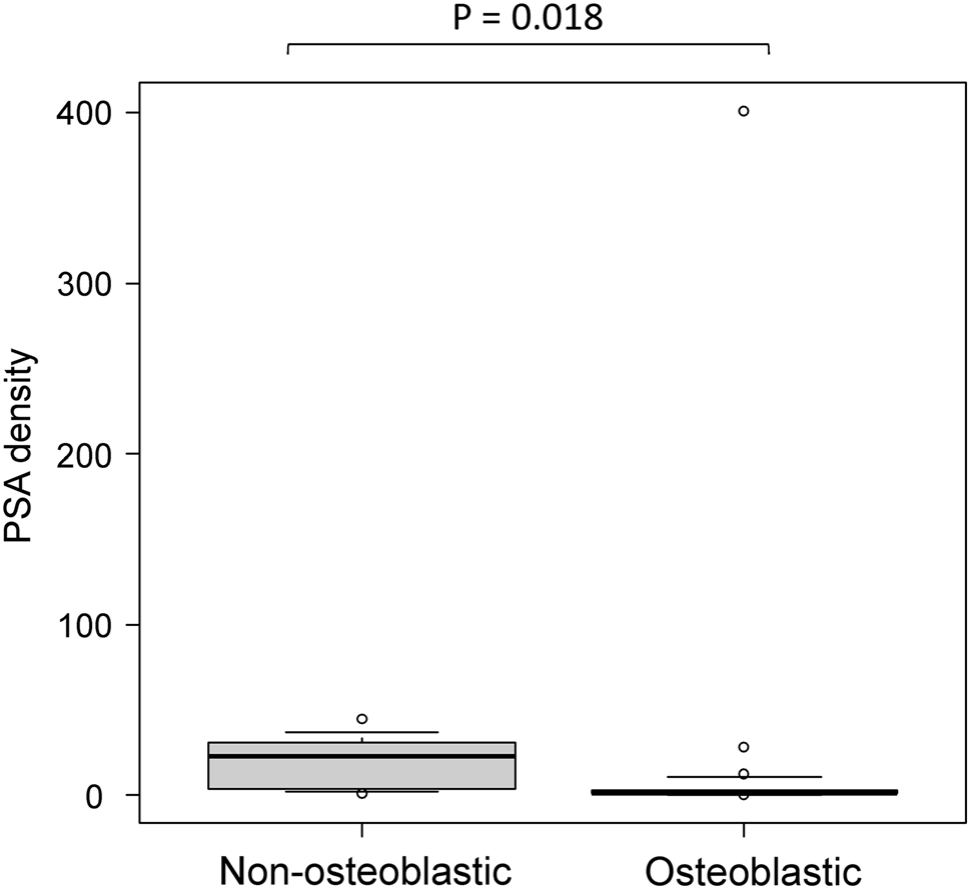
Comparison of PSA density between the osteoblastic group and non-osteoblastic group. The median and 10th and 90th percentiles are marked with boxplots.

**Figure 4:**
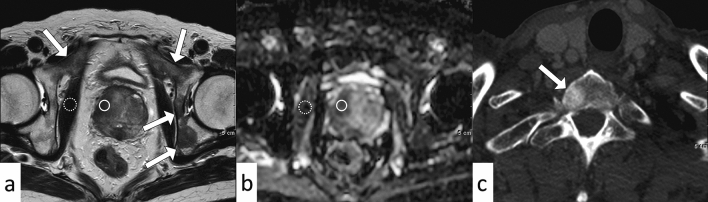
A 79-year-old man with prostate cancer with osteoblastic bone metastases (prostate-specific antigen density = 12.9). Prostate MRI shows the primary tumor (Gleason score 8) with a normalized T2 signal intensity of 2.7 (**a**, T2 signal intensity of prostate cancer (circle), divided by the T2 signal intensity of internal obturator muscle (dotted circle)), and normalized apparent diffusion coefficient of 0.67 (**b**, mean ADC of PCa (circle), divided by the ADC of the internal obturator muscle (dotted circle)). Multiple bone metastases are shown on the T2-weighted image (**a**, arrows). Axial CT reveals an osteoblastic lesion that is indicative of a bone metastasis (**c**, arrow).

**Figure 5:**
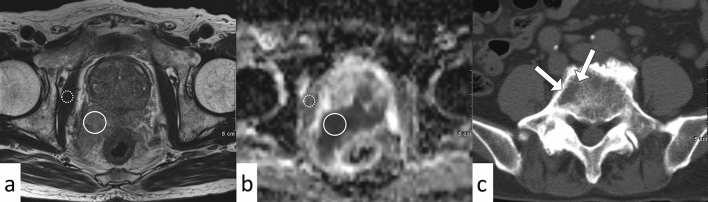
An 80-year-old man with prostate cancer with an osteolytic bone metastasis (prostate-specific antigen density = 5.6). Prostate MRI shows the primary tumor (Gleason score 9), with normalized T2 signal intensity of 3.2 (**a**) and normalized apparent diffusion coefficient of 0.50 (**b**). Axial CT reveals an osteolytic lesion that is indicative of bone metastasis (**c**, arrow).

**Figure 6:**
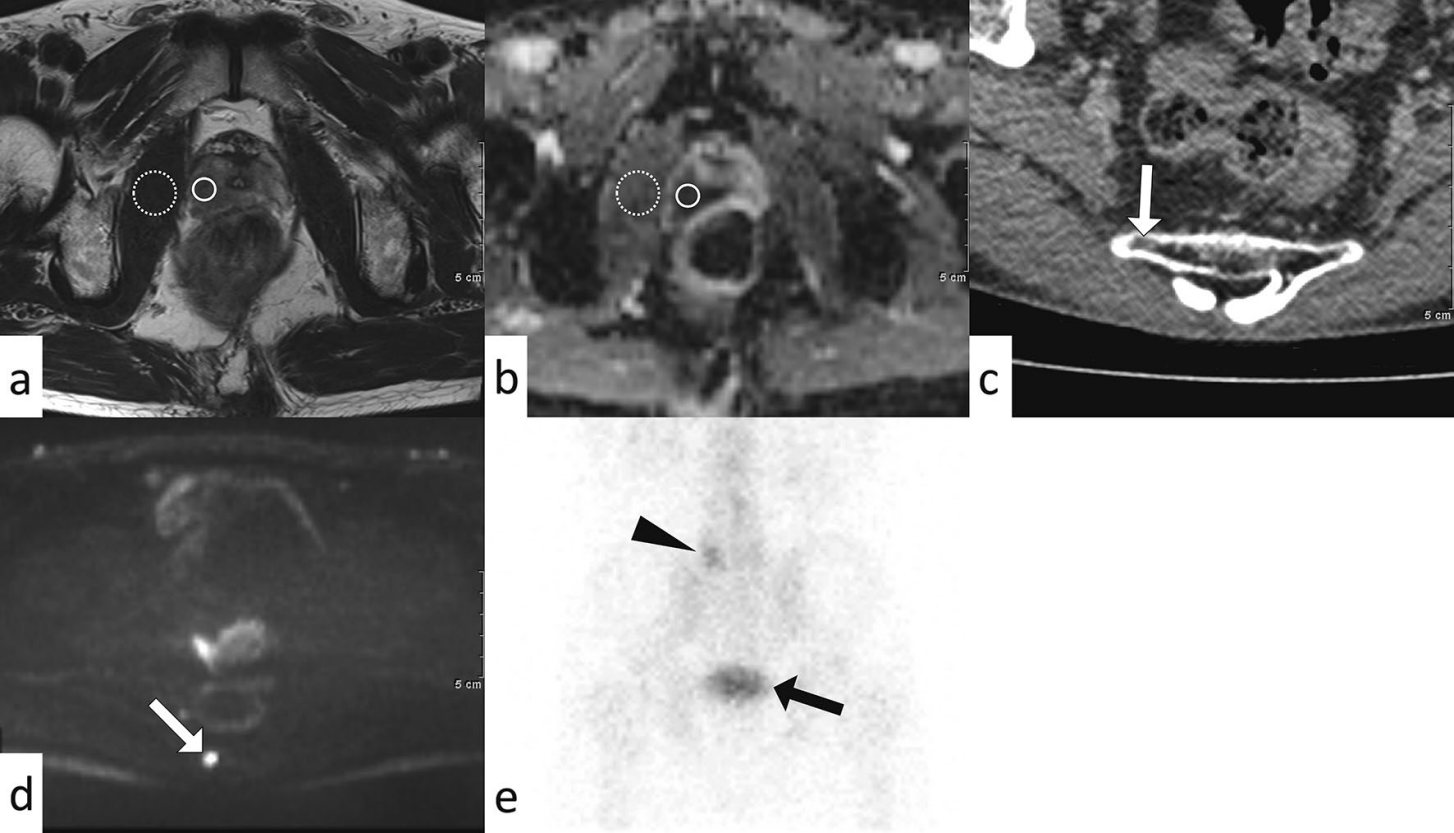
A 64-year-old man with prostate cancer with an osteolytic bone metastasis (prostate-specific antigen density = 2.5). Prostate MRI shows the primary tumor (Gleason score 9), with a normalized T2 signal intensity of 2.4 (**a**) and normalized apparent diffusion coefficient of 0.52 (**b**). CT shows an osteolytic bone metastasis in the sacrum (**c**, arrow), with hyperintensity on the diffusion-weighted image (**d**, arrow) but negativity on bone scintigraphy (**e**). The increased tracer uptake in the fifth lumbar vertebra is due to a degenerative change (**e**, arrowhead). The increased tracer uptake pointed by the arrow is the bladder (**e**, arrow).

The results of the clinical and radiological analyses are summarized in Table 1. The number of cases with BMs in the pelvis, vertebra, and other regions is summarized in Table 2. Prostate-specific antigen (PSA) density and normalized T2 signal intensity (nT2SI) were significantly higher in the non-osteoblastic group than in the osteoblastic group (PSA density: median 23.1 ng/mL/cm^3^ [range, 0.69–44.7] vs. 1.3 [0.076–401], *p* = 0.018; nT2SI: mean ± SD 3.3 ± 0.94 vs. 2.6 ± 0.61, *p* = 0.027). Figures 2 and 3 show the distributions of nT2SI and PSA density, respectively. No significant difference was found in age at diagnosis, Gleason score (GS), normalized mean apparent diffusion coefficient (nADCmean), or PI-RADS category between the two groups. Representative patients are shown in Fig. 4 (osteoblastic) and Figs. 5 and 6 (non-osteoblastic). A multivariate logistic regression analysis was performed using GS, PSA density, and nT2SI, and nT2SI was found to be an independent predictive factor for the non-osteoblastic group (*p* = 0.039).

**Table 1:** Clinical and radiological analyses.

	Total	Osteoblastic	Osteolytic & mixed	*P*-value	Statistical test statistic score
Number	32	25	7		
Age at diagnosis (median years, range)	72 (54–91)	72 (83–65)	65 (54–91)	0.19	Mann–Whitney U test U = 116.5
Gleason score (median, range)	8 (6–10)	8 (6–10)	9 (8–9)	0.11	Mann–Whitney U test U = 56
PSA density (median ng/mL/cm^3^, range)	1.6 (0.076–401)	1.3 (0.076–401)	23.1 (0.69–44.7)	0.018*	Mann–Whitney U test U = 36
Normalized mean ADC (median, range)	0.87 (0.46–7.7)	0.93 (0.46–7.7)	0.59 (0.50–1.6)	0.35	Mann–Whitney U test U = 109
Normalized T2 signal intensity (median, range)	2.7 (1.0–4.7)	2.6 (1.0–3.5)	3.1 (2.3–4.7)	0.027*	Student’s t-test t = 2.33
PI-RADS category (median, range)	5 (3–5)	5 (3–5)	5 (5–5)	0.65	Mann–Whitney U test U = 84

*Statistically significant.

*PSA* prostate-specific antigen, *ADC* apparent diffusion coefficient, *PI-RADS* prostate imaging reporting and data system.

**Table 2:** The number of cases with bone metastasis in each region.

	Pelvis	Vertebra	Others
Osteoblastic	20/25	15/25	17/25
Osteolytic & mixed	5/7	7/7	5/7

## Discussion

In this study, we investigated the differences in pretreatment prostate MRI and clinical findings between patients with PCa with osteoblastic and non-osteoblastic BMs. The PSA density and nT2SI were significantly higher in the non-osteoblastic group than in the osteoblastic group. The multivariate logistic regression analysis showed that nT2SI was an independent predictive factor in the non-osteoblastic group. In contrast, no significant difference was found in the GS, nADCmean, or PI-RADS categories between the two groups.

Prostate MRI has been widely used to diagnose and stage PCa in clinical practice^7–11^, and PI-RADS v2.1 was developed to improve risk stratification in patients with suspected PCa^6^. Assignment of PI-RADS category is based on multiparametric MRI findings including lesion size and extraprostatic extension, and good performance for the diagnosis of clinically significant PCa is reported^8^. The BMs of PCa have been considered predominantly osteoblastic; however, some PCa develop osteolytic or mixed BMs^5^. Differentiating osteolytic BMs from other osteolytic lesions, such as multiple myeloma or metastases from other malignancies, is clinically relevant^4^ given that the type of BMs influences lesion management^19^. Understanding certain MRI findings of PCa that are more likely to develop into osteolytic BMs may help prevent the overlooking of systemic BMs on CT. Conversely, it is important to not overlook the possibility that PCa can be the primary tumor when osteolytic BMs are detected on CT. To our knowledge, this is the first study to evaluate and compare prostate MRI findings between different types of BMs.

PSA density is a useful predictor for local invasion, lymph node metastasis, and biochemical recurrence of PCa^20–23^. PSA density has also been reported to be a predictor of the presence of BMs^24^, although the type of BMs has not been evaluated. In this study, the PSA density was significantly higher in the non-osteoblastic group than in the osteoblastic group. PSA itself is known to promote osteoblastic changes^25^; however, the type of BMs is influenced by multifactorial mechanisms and complex tumor-bone interactions^26^. Non-osteoblastic BMs are more frequent in high-risk PCa^12^; therefore, high PSA density in the non-osteoblastic group in this study may reflect the aggressiveness of PCa, although the difference in GS did not reach statistical significance, possibly because of the overlap between the groups.

Peker et al. reported that nT2SI was significantly higher in the prostatitis group than in the PCa group^27^, and Wang et al. reported that nT2SI negatively correlated with GS^18^. T2 has been reported to negatively correlate with cell density, percentage area of nuclei, and percentage area of cytoplasm and positively correlate with the percentage area of luminal space^13,28^. In this study, nT2SI was significantly higher in the non-osteoblastic group than in the osteoblastic group, and was found to be an independent factor for predicting the non-osteoblastic group in the multivariate logistic regression analysis. Furthermore, the GS tended to be higher in the non-osteoblastic group than in the osteoblastic group, although the difference was not statistically significant. The results of the present study indicate that higher nT2SI despite high GS may predispose patients to non-osteoblastic BMs. This finding needs to be validated in a larger study.

Apparent diffusion coefficient (ADC) is one of the factors for determining the PI-RADS category^6^ and negatively correlates with GS and cell density^13–17^. The association between ADC and D'Amico clinical risk scores and the Ki-67 positivity rate of PCa has also been reported^16,17^. However, no significant difference was observed in the nADCmean between the osteoblastic and non-osteoblastic groups in this study. PCa with non-osteoblastic BMs may have a combination of characteristics that raise and lower ADC, such as low cell density for high GS. Further studies with a larger number of patients are necessary to validate these findings.

Bone scintigraphy using 99mTc-diphosphonates is the most widely available imaging modality for detecting BMs in patients with PCa. However, up to 50% of osteolytic BMs cannot be detected^29^. In fact, BMs that are negative on bone scintigraphy and positive on DWI have been observed, as reported in the present study (Fig. 6). Therefore, patients with PCa with high nT2SI and PSA density should be examined with osteolytic BMs in mind, which can be negative on bone scintigraphy.

In accordance with PI-RADS ver. 2.1^6^, T2-weighted images and diffusion-weighted images were evaluated in this study. Due to the limited size of the study population, we did not include contrast-enhanced dynamic T1-weighted imaging in this study. Further studies using contrast-enhanced dynamic T1-weighted imaging with more patients are needed. Additionally, the correlation between MRI findings and histological findings or gene expression and their usefulness as biomarkers, including their combinations, are important issues to be addressed in the future. Imaging biomarkers would offer the opportunity to advance precision diagnostics and enable better management of PCa. Since many patients with PCa are long-term survivors, a large size of study population with a long-term investigation is required for survival analysis to validate their usefulness as biomarkers.

This study has some limitations. First, this was a single-institution retrospective study that included a small number of patients. Second, none of the BMs was pathologically proven, and the possibility of BMs from other malignancies was not ruled out. However, this possibility was mitigated by excluding patients with concomitant or past malignancies. Finally, ADC and T2SI could have been affected by different MRI scanners and imaging parameters. However, we performed normalization for these parameters to mitigate the effects.

In conclusion, comparison of MRI and clinical findings between the osteoblastic and non-osteoblastic BMs groups of patients with PCa revealed that nT2SI and PSA density were significantly higher in the non-osteoblastic group. nT2SI was identified as an independent predictive factor for non-osteoblastic BMs in the multivariate logistic regression analysis. These results indicate that patients with PCa with high nT2SI and PSA density should be examined for osteolytic BMs.

## Methods

The study was approved by the ethics committee of the Research Ethics Committee of the Faculty of Medicine of the University of Tokyo (Approval Number 2561-(23)). The need for informed consent was waived by the ethics committee of the Research Ethics Committee of the Faculty of Medicine of the University of Tokyo due to the retrospective study design. Data were acquired in compliance with all applicable regulations of the Health Insurance Portability and Accountability Act and all methods were performed in accordance with relevant guidelines and regulations. Data were de-identified prior to any analysis.

### Patients

We performed a patient search using our hospital’s database. Between 2014 and 2021, 2314 patients with pathologically proven PCa were identified in our hospital database. The following inclusion and exclusion criteria were used for patient selection. Some of the patients were evaluated in the previous study^12^

Inclusion criteria:

- Prostate MRI performed within six months before pathological diagnosis
- Body CT performed within six months before or after pathological diagnosis of PCa, and BMs were detected on CT examination

Exclusion criteria:

- Patients with concomitant or history of malignancy other than PCa
- Treatment started prior to prostate MRI
- BMs detected on CT after the beginning of treatment

### Imaging acquisition

All body CT scans were performed using 16–320-row multi-detector CT units. The scanning protocol included an unenhanced and/or contrast-enhanced phase (delay time: 90–120 s) after intravenous administration of 600–740 mg iodine/kg body weight of iodine contrast agent at 1.0–3.3 mL/sec, using an automated power injector. Images were reconstructed at a 5.0 mm thickness on the axial plane. The scanning parameters included a field-of-view to fit (300–420 mm), 512 × 512 matrix, 120 kVp, and 110–700 mA.

MRI was performed using 1.5 T or 3.0 T scanners. T2-weighted images were acquired using the following parameters: turbo spin echo; repetition time, 2000–20,000 ms; echo time 79–203 ms; flip angle of 90–160°; and resolution, 0.312 × 0.312–0.938 × 0.938 mm^2^. Diffusion-weighted images were acquired using the following parameters: repetition time, 3209.3–16,300 ms; echo time, 59.2–95 ms; resolution, 0.885 × 0.885–3.017 × 3.017 mm^2^; and b values, at least (0 or 50 s/mm^2^) and (800, 1000, or 1500 s/mm^2^). The ADC maps were calculated from the raw data.

### Clinical and radiological data

The following data were collected from the medical records of the patients:

- Age at pathological diagnosis of PCa
- GS
- Type of BMs on CT (osteoblastic, osteolytic, or mixed)
- PSA density, nADCmean, and nT2SI

PSA density was defined as PSA divided by prostate volume, which was calculated as width × height × length × 0.52^21^. nADCmean was calculated by dividing the mean ADC of PCa by the mean ADC of the internal obturator muscle^30^. The nT2SI was calculated by dividing the mean T2 signal intensity of the PCa by the mean T2 signal intensity of the internal obturator muscle^18^.

### Imaging analysis

All CT examinations were analyzed by consensus, by an experienced board-certified radiologist and a training radiologist. They were blinded to the patients’ clinical and pathological information, except that they were diagnosed with PCa. They evaluated the type of BMs on CT images, and classified BMs with only sclerotic component as osteoblastic, BMs with only lytic component as osteolytic, and BMs with both components as mixed. The prostate size for calculating PSA density, nADCmean, and nT2SI of PCa were evaluated using MRI. ADC and T2SI were measured by placing circle region-of-interest, as large as possible, on the targeted areas while carefully avoiding the rim and vessels.

### Statistical analysis

Patient age, GS, PSA density, nADCmean, and nT2SI were compared between the osteoblastic and non-osteoblastic BM groups using Student’s t-test or the Mann–Whitney U test, depending on the results of Shapiro–Wilk tests. A multivariate logistic regression analysis using factors with *P*-values < 0.2 was performed to detect the independent parameters for predicting the non-osteoblastic BM group. Statistical significance was set at *p* < 0.05. All statistical analyses were performed using the R software (version 4.0.3; R Foundation for Statistical Computing, Vienna, Austria).

## Data Availability

The datasets generated and analyzed during the current study are available from the corresponding author on reasonable request.
